# Supplementary material for: In Vitro Assessment of Hydrolysed Collagen Fermentation Using Domestic Cat (Felis catus) Faecal Inocula
Source: Animals (Basel). 2022 Feb 17;12(4):498. doi: 10.3390/ani12040498 (PMC8868200; doi:10.3390/ani12040498)
Supplement: Supplementary file 1 [file animals-12-00498-s001.zip › animals-1534653-supplementary.pdf]

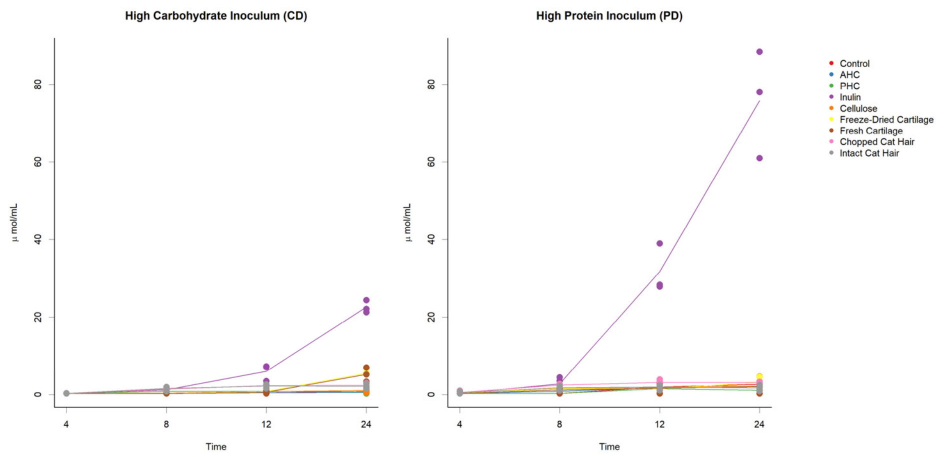

Figure S1: Scatter plot and means of lactate concentrations. Time (hours) is along the x-axis and organic acid concentration along the y-axis (mM). Both high protein faecal inoculum (PD) and high carbohydrate faecal inoculum (CD) and the changes that occurred over 24 h of fermentation are shown. Each point (coloured circle) represents an individual replicate, and each line shows the mean for each substrate. Control samples are denoted by a black solid line, AHC (ANZCO hydrolysed collagen) by a red dashed line, PHC (Peptan hydrolysed collagen) by a blue dashed line, cellulose by a green dashed line, inulin by an orange dotted line, freeze-dried cartilage by a brown dot-dashed line, fresh cartilage by a purple large dashed line, chopped cat hair by a pink dot-dashed line, and intact cat hair by a light blue dot double-dashed line.
